# Adsorptive Stripping Voltammetric Quercetin Determination in Pharmaceuticals and Urine Samples Using a Long Service-Life Array of Carbon Composite Microelectrodes

**DOI:** 10.3390/molecules29184464

**Published:** 2024-09-20

**Authors:** Iwona Gęca, Mieczyslaw Korolczuk

**Affiliations:** Institute of Chemical Sciences, Faculty of Chemistry, Maria Curie Sklodowska University, 20-031 Lublin, Poland; mieczyslaw.korolczuk@mail.umcs.pl

**Keywords:** quercetin, the array of carbon composite microelectrodes, adsorptive stripping voltammetry, determination

## Abstract

This article presents for the first time a new working electrode with a long service life— the bismuth-plated array of carbon composite microelectrodes for the simple, fast and sensitive determination of quercetin by adsorptive stripping voltammetry. The main experimental conditions were selected. The calibration graph was linear from 1 × 10^−9^ to 2 × 10^−8^ mol L^−1^ with an accumulation time of 60 s. The detection limit was equal to 4.8 × 10^−10^ mol L^−1^. The relative standard deviation for 2 × 10^−8^ mol L^−1^ of quercetin was 4.4% (n = 7). Possible interference effects resulting from the presence of other organic and surface active compounds and interfering ions were studied. The developed procedure was successfully applied to determine quercetin in pharmaceutical preparations and the spiked urine samples.

## 1. Introduction

Flavonoids are a group of polyphenolic compounds commonly found in vegetables and fruits. These are compounds with antioxidant and anti-aging properties used on a large scale for various health and pharmacological purposes. Quercetin belongs to the flavonoids group and is reported to be an antitumor, anti-inflammatory, antiallergic, antibacterial and antiviral active [1,2,3]. The great interest of quercetin determination in various samples, e.g., in food, pharmaceuticals and urine, is connected with its potential activity in preventing and treating many diseases and its application in pharmacology. According to the literature data [4], the concentration of quercetin in real samples exhibits considerable variability. The observed range may span from about 1 × 10^−4^ to about 9000 µg g^−1^, depending on the specific sample under examination.

Quercetin has been reported to be determined by various analytical methods. Among them, the following methods can be found in the literature data: high-performance liquid chromatography (HPLC) [5,6,7,8], a combination of high-performance liquid chromatography and mass spectrometry (HPLC-MS) [9,10], UV-Vis spectrophotometry [11,12], spectrofluorimetry [13] and electrophoresis [14].

Quercetin was also reported to be determined by stripping voltammetry. This analytical method is competitive for the methods mentioned above, especially because of the low instrumentation costs and relatively short measurement time. The important factor that has an impact during voltammetric measurements is the selection of a working electrode to determine the particular analyte. The possibility of electroanalytical determinations under given pH conditions depends on the physical and chemical properties of the electrode material, the electrode surface or the film coated on its surface, and the possible operational potential window of a given working electrode. Moreover, the type of working electrode affects the quality of analytical results, e.g., the value of the detection limit and the separation of the obtained analytical signals. To date, a variety of working electrode materials and their constructions have been employed to determine quercetin in a range of samples. Among these, the following were proposed: carbon paste (nujol/graphite) electrode [3,15], carbon paste (paraffin oil) electrode [16], multi-wall carbon nanotube paste electrode [17,18,19,20]; variously modified glassy carbon electrode with single-walled carbon nanotubes [21], Nafion and carbon nanotubes [22], multi-wall carbon nanotubes dispersed in polyethylenimine and polyacrylic acid [23], flowerlike Co_3_O_4_ nanoparticles [24], siloxane-polyester/poly-L-lysine nanocomposite [25], carbon nanofibers and polythymolphthalein [26], platinum-polydopamine-coated silica particle nanocomposite [27], graphene oxide/silver nanoparticles [28] or poly (safranine O) [29]. Other modifications of the working electrodes employing, e.g., cyclodextrins, graphene oxide, ionic liquids or metals’ nanoparticles, and more or less complicated materials modifications were also reported [30,31,32,33,34,35,36,37].

A specific category of working electrodes employed in voltammetric measurements is a group of microelectrodes. The defining feature of microelectrodes is their minute size, which is on par with the thickness of the diffusion layer. The following advantages can be attributed to this configuration of working electrodes [38,39,40,41]: (i) low capacity of the electrical double layer; (ii) due to the occurrence of spherical diffusion at the microelectrode surface, it is possible to undertake the measurements from unmixed solutions that offer the prospect of the streamlined measurements and the capacity of conducting the measurements in field conditions; (iii) the signal-to-noise ratio is more favorable when working with microelectrodes as compared to the conventionally sized electrodes; (iv) the possibility of analyzing samples of a very small volume, from solutions with a low concentration of a supporting electrolyte and solutions of organic solvents are further advantages of using microelectrodes. On the other hand, the limited surface area of the microelectrodes results in the recording of low currents that are susceptible to interference. To address this limitation, assemblies and arrays comprising tens or hundreds of microelectrodes are constructed to enhance recorded currents and their resilience to interference.

To the best of our knowledge, there are very few reports in the literature describing the use of microelectrodes for the determination of quercetin. In [42], the authors applied a hot platinum microelectrode to determine flavonoids via flow injection analysis and capillary electrophoresis using electrochemical detection. In another work [43], a glassy carbon microelectrode of a diameter of 0.7 mm was used to study the oxidation mechanism of quercetin in nonaqueous media. Nevertheless, in consideration of the established definition of microelectrodes [40], the dimensions of the working electrode used in the referenced work many times exceed the generally accepted dimensional standards.

The objective of this article is to present a methodology for the determination of quercetin in pharmaceutical and urine samples, employing an array of carbon composite microelectrodes electrochemically modified with a bismuth film. The proposed array of carbon composite microelectrodes offers a significant advantage in terms of its long service life, which results from the way it is constructed. In contrast to the aforementioned articles, the modification of the surface of this sensor for quercetin determination is simple, uncomplicated and fast, and it consists only of the electrochemical preplating of a bismuth film during the standard measurement procedure. The microelectrode properties of the proposed sensor and assessment of the interaction between diffusion layers of individual microelectrodes were examined by cyclic voltammetry. After the optimization of the voltammetric procedure and the development of analytical characteristics, the voltammetric procedure was used for the analysis of pharmaceuticals and spiked urine samples. The obtained results confirm the possibility of determining quercetin in real samples. The comprehensive results of a voltammetric procedure optimization for the determination of quercetin are presented in the following sections.

## 2. Discussion

### 2.1. Microelectrode Properties of the Array of Carbon Composite Microelectrodes

**Figure 1:**
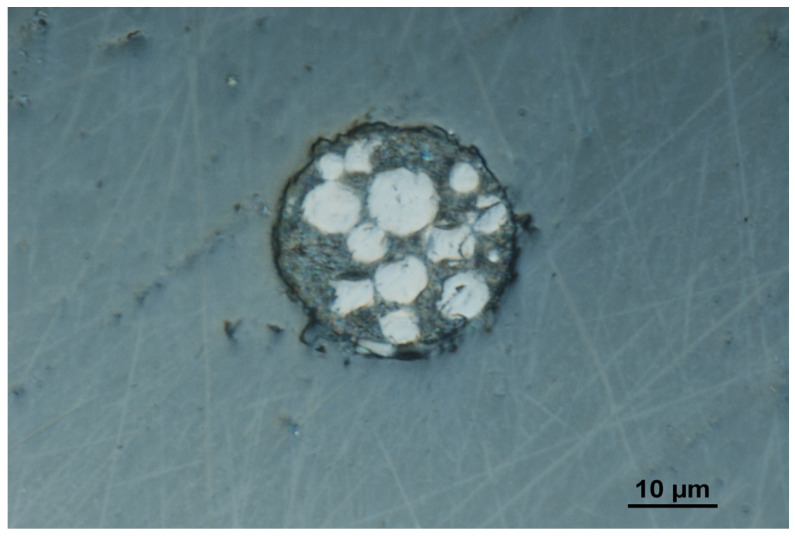
A real image of a single microelectrode from the array of carbon composite microelectrodes.

A real view of the surface of a single microelectrode from the array of carbon composite microelectrodes is presented in Figure 1.

**Figure 2:**
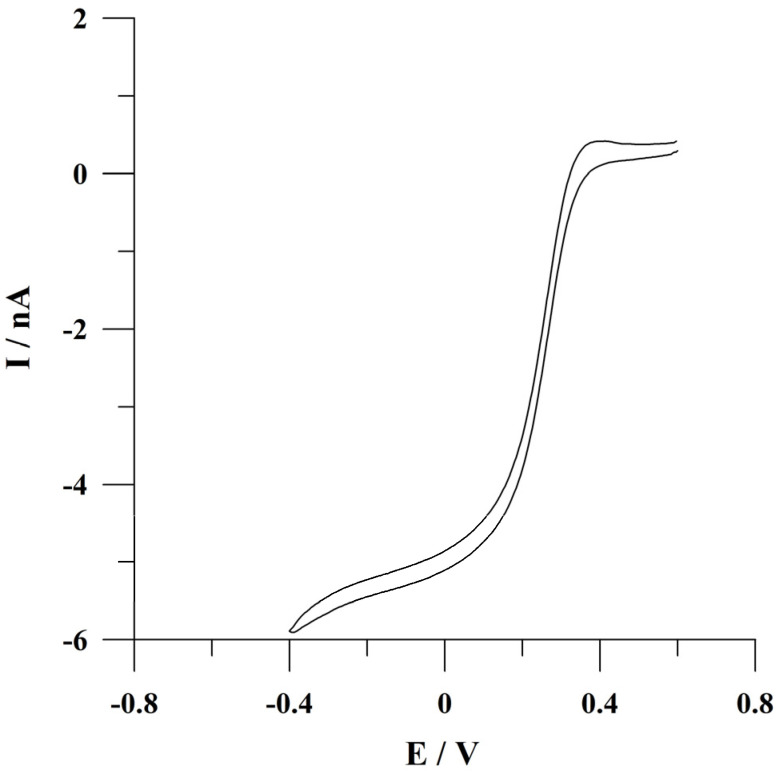
Cyclic voltammogram recorded using the array of carbon composite microelectrodes from a solution containing 1 mol L^−1^ KCl and 1 × 10^−3^ mol L^−1^ K_3_Fe(CN)_6_. Scan rate: 10 mV s^−1^.

To assess the microelectrode properties of the applied small-sized working electrode, a cyclic voltammogram was recorded from a solution containing 1 mol L^−1^ KCl and 1 × 10^−3^ mol L^−1^ K_3_Fe(CN)_6_ using cyclic voltammetry at low scan rate values. The obtained cyclic voltammogram is presented in Figure 2. Based on the presented sigmoidal shape of a cyclic voltammogram, it can be concluded that microelectrode properties characterize the proposed array of carbon composite microelectrodes. The obtained results also allow for assessing the interaction between diffusion layers of individual microelectrodes in the array and indicate the occurrence of hemispherical diffusion layers on individual microdisks. These observations are confirmed by data in the literature [44].

### 2.2. Optimization of the pH of the Supporting Electrolyte

**Figure 3:**
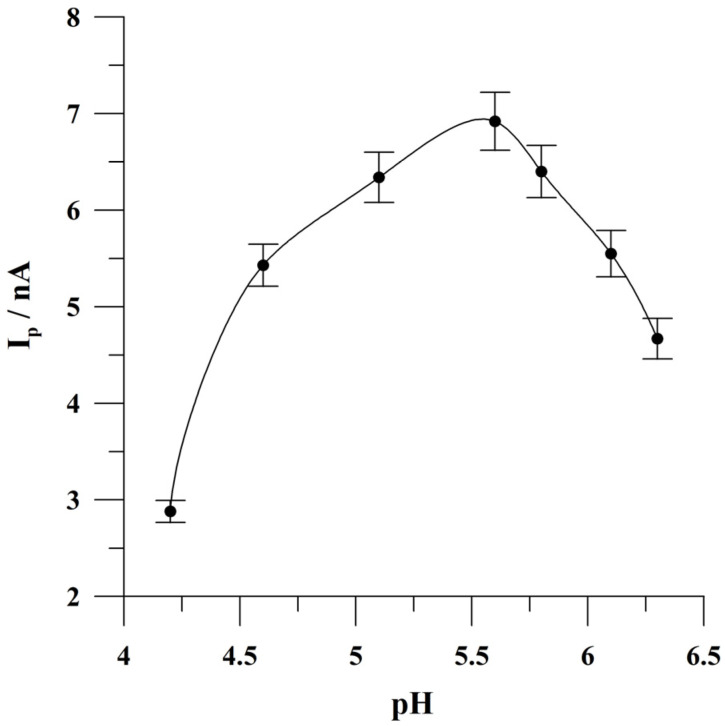
The influence of the pH of the supporting electrolyte on quercetin peak current. Concentration of quercetin was 5 × 10^−8^ mol L^−1^. Accumulation conditions: −0.45 V, 60 s. The error bars represent the standard deviation (n = 3).

The impact of the pH of the supporting electrolyte was examined in the range from 4.2 to 6.3 for a solution containing 5 × 10^−8^ mol L^−1^ of quercetin. The pH of the supporting electrolyte was adjusted by the addition of the appropriate quantity of either acetic acid or sodium hydroxide. The obtained results are presented in Figure 3. It was found that quercetin’s peak current increased to the pH value of 5.6 and then decreased at higher pH levels. The efficiency of quercetin accumulation on the electrode surface can be significantly influenced by the form of protonated species of quercetin present at a given pH because, as reported previously [45,46], the content of quercetin species strongly depends on the pH. For further studies, a pH of 5.6 was chosen. It is worth noting that the possibility of bismuth film plating at such a pH value without observing the hydrolysis of Bi(III) ions results from the presence of potassium-sodium tartrate in the supporting electrolyte. The addition of this reagent stabilizes bismuth ions in a weakly acidic and slightly alkaline solution, as reported previously [47,48].

### 2.3. Optimization of a Concentration of Bi(III)

**Figure 4:**
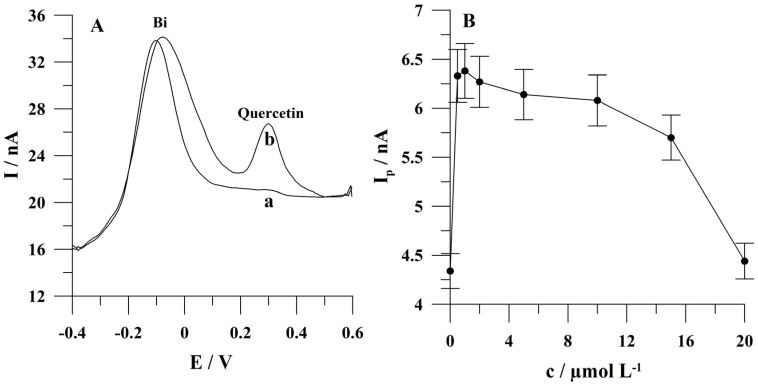
(**A**) Stripping voltammograms obtained using a carbon composite microelectrode array modified with bismuth film from a solution containing the following: (a) 2 × 10^−5^ mol L^−1^ Bi(III); (b) as (a) + 5 × 10^−8^ mol L^−1^ quercetin. *(***B**) The influence of a concentration of Bi(III) ions on quercetin peak current. Concentration of quercetin was 5 × 10^−8^ mol L^−1^. Accumulation conditions: −0.45 V, 60 s. The error bars represent the standard deviation (n = 3).

The proposed voltammetric procedure of quercetin determination is based on quercetin adsorptive accumulation at a carbon composite microelectrode array modified with the bismuth film and then quercetin oxidation during the stripping step. The adsorption mechanism of quercetin, its electrochemical oxidation on the surface of a glassy carbon electrode and a possible electrode mechanism were described previously [49,50]. During preliminary experiments, quercetin’s well-shaped oxidation analytical signal was obtained using a carbon composite microelectrode array modified with the bismuth film. The stripping voltammograms recorded during the initial experiments are presented in Figure 4A. The presented results indicate both bismuth deposition on the surface of a carbon composite microelectrode array and quercetin accumulation on a bismuth-modified carbon composite microelectrode array.

The impact of Bi(III) concentration on the quercetin peak current was studied for a solution not containing bismuth ions and for a solution containing these ions in the concentration range from 5 × 10^−7^ to 2 × 10^−6^ mol L^−1^. The quercetin concentration was 5 × 10^−8^ mol L^−1^. The results obtained during these investigations are presented in Figure 4B. Based on the results obtained, the following conclusions can be drawn.

The first one is that quercetin is accumulated at the unmodified surface of carbon composite microelectrode array; however, the addition of Bi(III) enhances the effectiveness of quercetin accumulation. It was found that the quercetin peak current increased by about 30% after the addition of bismuth ions in comparison to the case of these ions’ absence.

Next, it was found that quercetin’s peak current increased from 0 to 1 × 10^−6^ mol L^−1^ and then decreased at higher bismuth ion concentrations. As can be seen in Figure 4B, for low Bi(III) ion concentrations, the accumulation of quercetin rapidly increases. The effectiveness of the accumulation of analytes on a bismuth film electrode depends on its morphology [51,52]. According to the mentioned literature data, the thickness and compactness of the bismuth layer mainly depend on the composition of plating media, the potential of deposition and the concentration of Bi(III) [51,52]. At low Bi(III) concentrations, bismuth is deposited on the electrode in agglomerate forms. The edges of these agglomerates exhibit the greatest electrochemically active properties, resulting in an observable increase in the quercetin signal. At high Bi(III) concentrations, the edges of the agglomerates gradually diminish resulting in a decrease in the intensity of the quercetin signal. In conclusion, in the case of quercetin determination, a low concentration of Bi(III) in solution is preferable for further effective accumulation of the analyte on the electrode. An alternative hypothesis to explain the observed decrease in the peak current of quercetin at higher Bi(III) concentrations is that partial hydrolysis of Bi(III) ions may result in the co-precipitation of quercetin on bismuth hydroxide, thereby reducing the analytical signal of quercetin. Further studies were conducted in the presence of 1 × 10^−6^ mol L^−1^ of Bi(III).

The next important observation was that in the case of the absence of Bi(III) ions, quercetin is insufficiently stripped from the surface of the working electrode, which was proven by the results of measurements conducted at 0 s of accumulation after the measurements followed by the standard procedure of measurements. In such a case, the analytical quercetin signal was observed. As a consequence, the repeatability of the obtained results was adversely affected. On the contrary, the addition of Bi(III) ions to the supporting electrolyte improved the stripping of quercetin from the working electrode surface. This fact can be explained by the simultaneous stripping of accumulated bismuth and quercetin. The repeatability of the obtained quercetin analytical signal expressed as RSD value for seven subsequent measurements at the bismuth-modified array of carbon composite microelectrodes was checked from a solution containing 5 × 10^−8^ mol L^−1^ of quercetin for 60 s of accumulation. The calculated RSD was 4.4%, which confirms a good repeatability of the measurements.

The facts described above confirm the benefits of the addition of Bi(III) ions into the supporting electrolyte solution in the case of quercetin determination using the array of carbon composite microelectrodes.

### 2.4. Optimization of the Potential of Bismuth Plating

**Figure 5:**
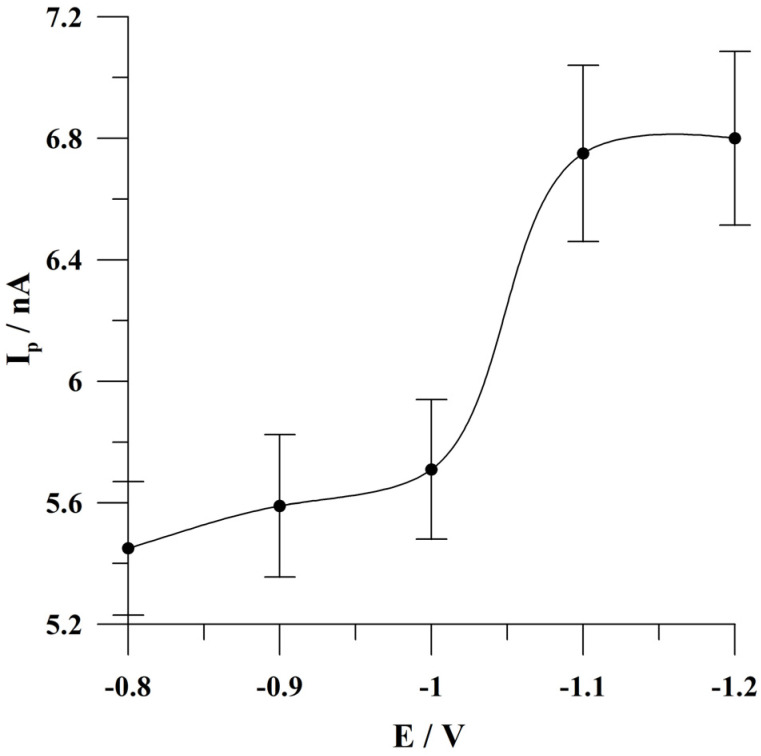
The influence of the potential of bismuth film preplating on quercetin peak current. Concentration of quercetin was 5 × 10^−8^ mol L^−1^. Accumulation conditions: −0.45 V, 60 s. The error bars represent the standard deviation (n = 3).

The influence of the potential of bismuth film preplating was studied in the range from −0.8 to −1.2 V. The time of the bismuth film preplating was 30 s. The accumulation conditions were −0.45 V, 60 s. The quercetin concentration was 5 × 10^−8^ mol L^−1^. The results obtained during these studies are presented in Figure 5. Based on these results, it can be concluded that the optimal potential value of bismuth preplating ranges from −1.1 to −1.2 V. Further investigations were conducted at the potential of −1.1 V because at more negative potential values, hydrogen reduction on the bismuth film may be observed.

### 2.5. Optimization of Quercetin Accumulation Conditions

**Figure 6:**
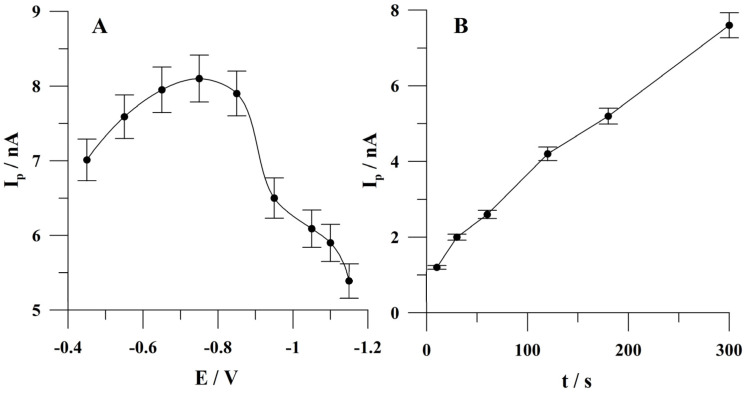
The influence of accumulation potential (**A**) and accumulation time (**B**) on quercetin peak current. Quercetin concentration: (**A**) 5 × 10^−8^ mol L^−1^; (**B**) 1 × 10^−8^ mol L^−1^. Accumulation conditions: 60 s (**A**); −0.75 V (**B**). The error bars represent the standard deviation (n = 3).

The next optimized parameter was the quercetin accumulation potential. The effect of accumulation potential was studied from −0.45 to −1.15 V. The accumulation time was 60 s. The concentration of quercetin was 5 × 10^−8^ mol L^−1^. The obtained results are shown in Figure 6A. The quercetin peak height increased from −0.45 V, attained the highest value at −0.75 V, and decreased at more negative accumulation potential values. Further studies were conducted at an accumulation potential of −0.75 V. It was observed that at an optimal value of the potential of bismuth preplating of −1.1 V quercetin was also accumulated; however, the effectiveness of its accumulation in these conditions was lower than at a potential of −0.75 V. The most efficient accumulation of quercetin was observed when the step of bismuth film plating at a more negative potential and the step of accumulating quercetin at a less negative potential were combined in one measurement cycle.

The impact of quercetin accumulation time was studied from 10 to 300 s. The quercetin concentration was 1 × 10^−8^ mol L^−1^. The obtained results are presented in Figure 6B. It was found that quercetin peak current increased almost linearly up to 300 s throughout the whole studied range of accumulation time. Further research was carried out to shorten the measurement time at 60 s of accumulation. However, based on the obtained results, it can be concluded that the lowest concentrations of quercetin can be determined at longer accumulation time.

### 2.6. Calibration Studies

**Figure 7:**
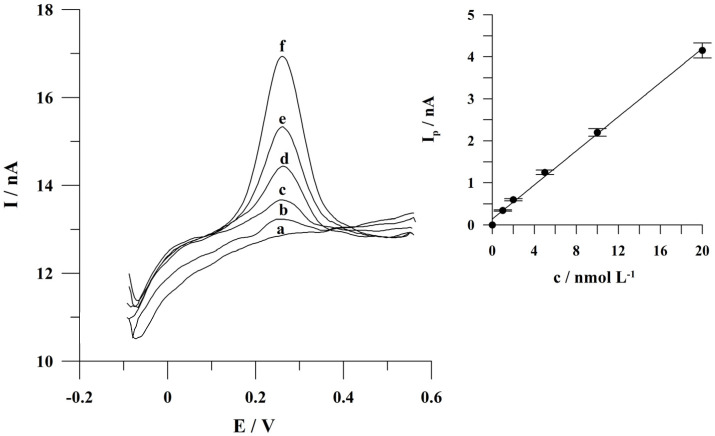
Adsorptive stripping voltammograms obtained for increasing quercetin concentrations and the corresponding linear calibration graph as an inset: Quercetin concentrations: (a) 0; (b) 1 × 10^−9^; (c) 2 × 10^−9^; (d) 5 × 10^−9^; (e) 1 × 10^−8^; (f) 2 × 10^−8^ mol L^−1^. Accumulation conditions: −0.75 V, 60 s. The error bars represent the standard deviation (n = 3).

Under the optimized conditions, the calibration graph of quercetin for an accumulation time of 60 s was linear from 1 × 10^−9^ to 2 × 10^−8^ mol L^−1^ and obeyed the equation y = 0.19x + 0.13, where y and x are the peak current (nA) and the quercetin concentration (nmol L^−1^), respectively. The linear correlation coefficient r was 0.997. The relative standard deviations from seven subsequent determinations of quercetin at a concentration of 2 × 10^−8^ mol L^−1^ was 4.4%. The detection limit estimated as three times the standard deviation of intercept divided by the slope of the calibration plot was equal to 4.8 × 10^−10^ mol L^−1^ for 60 s of accumulation. Voltammograms obtained for increasing quercetin concentrations during calibration studies for an accumulation time of 60 s and the corresponding calibration graph are presented in Figure 7. It should be stated that the range of quercetin concentrations to be determined can be extended by lengthening or shortening the accumulation time.

### 2.7. Reproducibility Studies

The stability in time of the proposed carbon composite microelectrode array expressed as the reproducibility of the quercetin analytical signal was examined for the supporting electrolyte containing 2 × 10^−8^ mol L^−1^ of quercetin by conducting seven consecutive measurements of the sample about one year after the procedure was developed. The obtained RSD value was equal to 5.3%.

Moreover, the reproducibility of the quercetin analytical signal was studied by comparing the results obtained for five carbon composite microelectrode arrays prepared in a way described in the Instrumentation section. The RSD calculated from the results obtained during this study was equal to 4.9%.

The obtained results indicate satisfactory reproducibility and long service-life properties of the described carbon composite microelectrode arrays.

### 2.8. Interferences Studies

The potential interferences that can be observed during an analysis of the pharmaceutical and urine samples, including inorganic ions and organic substances, were studied from the solutions containing 2 × 10^−8^ mol L^−1^ of quercetin. The accumulation time was 60 s. The obtained results are presented in Table 1. The majority of the interfering ions and organic substances did not seriously affect the analytical signal of quercetin even at a high excess of studied interferents, so it can be concluded that the proposed voltammetric procedure is of high selectivity.

### 2.9. Analytical Application

**Figure 8:**
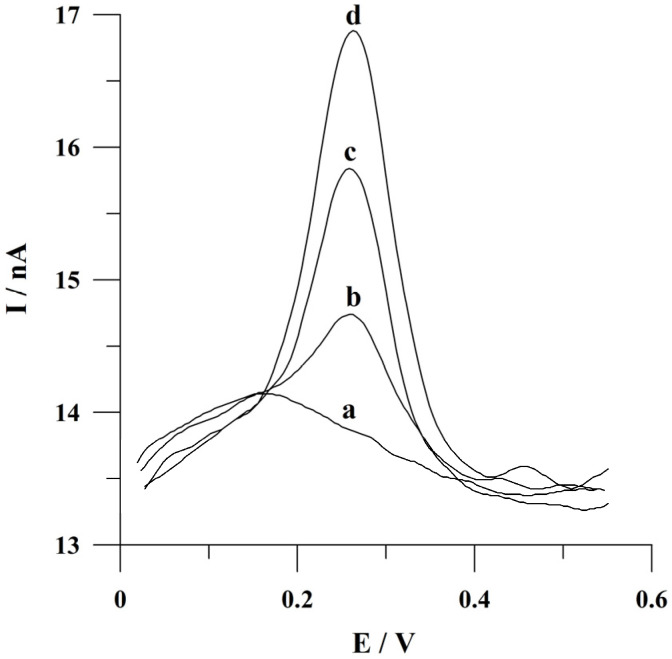
Adsorptive stripping voltammograms obtained during pharmaceutical formulation analysis: (a) supporting electrolyte; (b) supporting electrolyte with pharmaceutical preparation; (c) as (b) + 4 × 10^−9^ mol L^−1^; (d) as (c) + 4 × 10^−9^ mol L^−1^ of quercetin. Accumulation at −0.75 V within 60 s.

The described procedure of quercetin determination was used for an analysis of a pharmaceutical preparation. A sample of a volume of 4 µL prepared according to the procedure described in detail in the *Preparation of Pharmaceutical and Urine Samples* section was added to the supporting electrolyte in the electrochemical cell. The voltammetric analysis was carried out using the method of standard addition following the accumulation time of 60 s. The voltammograms obtained during the measurements are shown in Figure 8. The obtained result (the average quercetin mass per tablet) was 60.4 mg with a standard deviation of 4.6% (n = 5). The obtained result agreed well with the declared content of quercetin per tablet. Based on the obtained results, it can be concluded that the proposed voltammetric procedure of quercetin determination using the bismuth-plated array of carbon composite microelectrodes can be applied for the analysis of pharmaceutical formulations.

**Figure 9:**
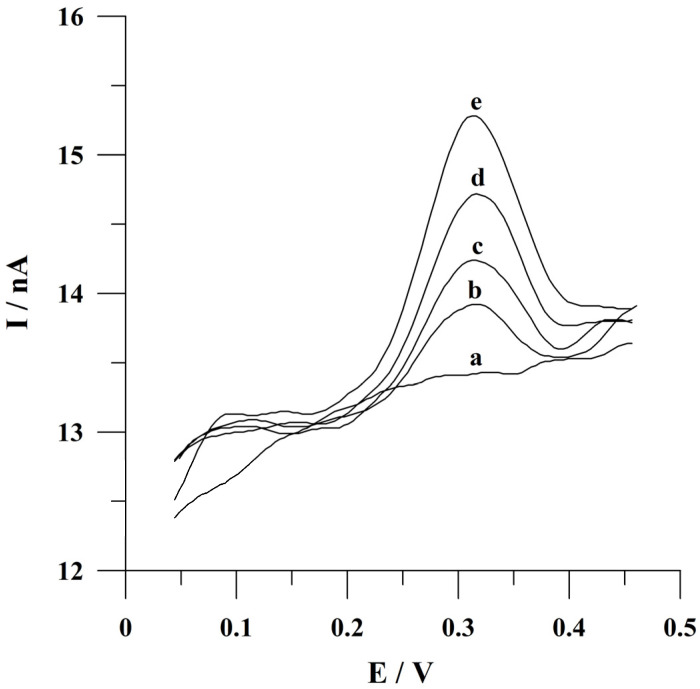
Adsorptive stripping voltammograms obtained during recovery studies from spiked urine sample: (a) the urine sample; (b) as (a) + 5 × 10^−9^ mol L^−1^; (c) as (b) + 5 × 10^−9^ mol L^−1^; (d) as (c) + 5 × 10^−9^ mol L^−1^; (e) as (d) + 5 × 10^−9^ mol L^−1^ of quercetin. Accumulation conditions: −0.75 V, 60 s.

Additionally, recovery studies were performed to check the developed procedure’s correctness by spiking the diluted and quercetin-free urine samples with fixed concentrations of quercetin. The factor of urine dilution was 200. Urine analysis was conducted employing the standard addition method at an accumulation time of 60 s. The obtained voltammograms are presented in Figure 9. Three replicate determinations of each sample containing 5 × 10^−9^ and 1 × 10^−8^ mol L^−1^ of quercetin resulted in average recovery values of 110 and 102% with a relative standard deviation (RSD) of 4.1 and 4.8%, respectively. The satisfying recovery values were obtained for quercetin determination in spiked urine samples. The described results indicated the fact that the proposed voltammetric procedure can be applied for quercetin determination in urine samples.

The results of the analysis of the pharmaceutical preparation and spiked urine samples are presented in Table 2.

## 3. Materials and Methods

### 3.1. Instrumentation

The experiments were performed using a μAutolab analyzer (from ECO/Chemie, Utrecht, The Netherlands). A traditional three-electrode configuration was used, with the array of carbon composite microelectrodes as a working electrode, an Ag/AgCl/NaCl reference electrode filled with saturated NaCl and a platinum wire counter electrode. An electrochemical cell of a volume of 10 mL was used. The working electrode was constructed as follows. Briefly, for the fabrication of the array of carbon composite microelectrodes, a homemade silica preform containing about 70 holes was used. Each hole has a nearly circular shape with a diameter of about 25 µm. The distance between holes was equal to about 200 µm. The holes in the silica preform were filled under pressure with a carbon composite paste prepared as follows. The composite was obtained by mixing carbon black (11%), glassy carbon spherical powder of a diameter of 2–12 mm (34%) and epoxy resin. The capillary filled with carbon composite was cured at a temperature of 110 °C for two days. After that, a 5 mm preform filled with composite was polished at both ends and placed in the Teflon housing of a diameter of 5 mm. Graphite powder was used for the electrical connection between microelectrodes and a copper wire. In such a way, a high-stability, long service-life working electrode was obtained. The surface of the microelectrode array was polished once a day directly before the voltammetric measurements using an abrasive paper of 2500 grit and then with paste from 0.3 µm Al_2_O_3_ on a Buehler polishing pad. After polishing, the electrode was cleaned with deionized water in an ultrasonic bath for about 30 s. A real view of the carbon composite microelectrode array was taken by MA200 Inverted Metallographic Microscope Nikon (Tokyo, Japan).

### 3.2. Reagents

A 1 mol L^−1^ acetate buffer of a pH of 5.6 was prepared from CH_3_COOH and NaOH, Suprapur reagents purchased from Merck. A 2 mol L^−1^ potassium sodium tartrate solution was prepared from the reagent obtained from Fluka (Buchs, Switzerland). A standard solution of bismuth at a concentration of 1 g L^−1^ was obtained from Fluka. Quercetin reagent was obtained from Sigma-Aldrich (St. Louis, MO, USA). A stock standard solution of quercetin at a concentration of 1 × 10^−3^ mol L^−1^ was prepared by dissolution of the reagent in ethanol and stored in a refrigerator until use. A quercetin working solution at a concentration of 1 × 10^−5^ mol L^−1^ was prepared daily by diluting the standard solution of quercetin in water. Reagents used for studying interferences were obtained from Sigma (St. Louis, MO, USA). A pharmaceutical preparation containing 60 mg of quercetin per tablet was used for investigating the accuracy of the procedure. All used chemical reagents were of analytical reagent grade or Suprapur. Lyophilized human urine was purchased from Medichem Diagnostica GmbH & Co. KG (Steinenbronn, Germany). Deionized water obtained from Milli-Q system purification was used for the preparation of all solutions.

### 3.3. Preparation of Pharmaceutical and Urine Samples

The developed voltammetric procedure was applied to the quercetin determination in pharmaceutical preparation. According to the data contained on the packaging, one effervescent tablet of this preparation contains 60 mg of quercetin, 600 mg of calcium and the following excipients: citric acid, sodium bicarbonate, calcium carbonate, sorbitol, polyvinylpyrrolidone, acesulfame K and aspartame. One tablet of this preparation was dissolved in 80 mL of ethanol to prepare a sample for analysis. Then, the extraction of quercetin was carried out for approximately 10 min in an ultrasonic bath to ensure a complete dissolution of quercetin. Next, 200 μL of the solution obtained was diluted in 50 mL of deionized water, and then appropriate amounts of the diluted sample solution were introduced into the electrochemical vessel. The preparation of the tablet was repeated on five occasions. The quercetin content in the analyzed preparation was determined using the standard addition method.

Urine samples were directly analyzed without any pretreatment apart from a dilution with a dilution factor of 200. Because the analyzed urine did not contain quercetin, the diluted and spiked urine samples were analyzed by conducting recovery studies using the standard addition method. The voltammetric analysis of quercetin-free and spiked samples was performed following a standard procedure of measurements described below. The obtained results of the urine sample analysis are presented further.

### 3.4. Standard Procedure of the Measurements

The analyzed sample was placed into the voltammetric cell and then 1 mL 1 mol L^−1^ of acetate buffer (pH 5.6), 1 mL 2 mol L^−1^ potassium-sodium tartrate and 2 μL 1 g L^−1^ Bi(III) were added and made up to 10 mL with deionized water. Potassium-sodium tartrate was used for stabilizing bismuth ions in weakly acidic pH conditions. The adsorptive stripping voltammetric measurements were conducted by applying the following potential sequence to the working microelectrode array: (i) electrochemical cleaning of the array of microelectrodes surface was conducted at the potential of 0.3 V within 30 s; (ii) then, bismuth film was mainly plated on the surface of array of microelectrodes at a potential of −1.1 V within 30 s; (iii) an accumulation of quercetin was carried out at potential of −0.75 V within 30 s. Then, after a rest ten-second equilibration period, a square wave voltammogram was recorded while the potential was changed from −0.2 to 0.6 V. The square wave parameters were as follows: frequency, 200 Hz; amplitude, 50 mV; step potential, 4 mV. The measurements were carried out without deoxygenation of the solution.

## 4. Conclusions

In this article, a new voltammetric working electrode—an array of carbon composite microelectrodes—was presented and used for pharmaceutical and biomedical analysis. The microelectrode properties of the proposed small-sized working electrode was proven by employing cyclic voltammetry. The proposed sensor, after its in situ modification with bismuth film, was applied for quercetin determination in pharmaceutical and spiked urine samples by adsorptive stripping voltammetry. The developed voltammetric procedure is characterized by high selectivity and precision as well as a low detection limit equal to 4.8 × 10^−10^ mol L^−1^ for 60 s of accumulation. The consistency of the results obtained during the pharmaceutical analysis with declared quercetin content, as well as the satisfying recovery values obtained during the urine sample analysis, confirm the correctness of the developed procedure. It is worth noting that thanks to the use of the developed analytical procedure, quercetin can be determined in samples with a complex matrix without complicated sample pretreatment.

## Figures and Tables

**Table 1:** Relative analytical signal of quercetin with and without the presence of interferents. Quercetin concentration was 2 × 10^−8^ mol L^−1^.

Interferent	A Molar Excess of Interferent	^1^ Relative Signal of Quercetin ± SD
K^+^	500	1.04 ± 0.037
Cl^−^	500	1.03 ± 0.041
Mg^2+^	500	1.05 ± 0.038
Ca^2+^	500	0.98 ± 0.046
NO_3_^−^	500	0.99 ± 0.043
NO_2_^−^	500	0.95 ± 0.039
Glucose	1000	1.1 ± 0.036
	10,000	0.91 ± 0.045
Folic acid	200	0.85 ± 0.044
Pyridoxine	200	0.97 ± 0.042
Ascorbic acid	500	0.98 ± 0.035
	10,000	1.07 ± 0.046
Urea	1000	1.02 ± 0.033
Hypoxanthine	1000	0.99 ± 0.042

^1^ Relative signal of quercetin—quercetin peak current ratio in the presence and absence of the interferent excess.

**Table 2:** The results of quercetin determination in the pharmaceutical preparation and spiked urine samples.

**Sample**	**Average Measured Content ± SD [mg]**	**Declared Value [mg]**	**Recovery ± RSD [%]**
Pharmaceutical preparation	60.4 ± 0.5	60.0	100.7 ± 0.9
**Sample**	**Added [nmol L^−1^]**	**Found average content ± SD [nmol L^−1^]**	**Recovery ± RSD [%]**
Urine sample	5.0	5.5 ± 0.2	110.0 ± 3.6
	10	10.2 ± 0.3	102.0 ± 3.0

## Data Availability

Data are contained within the article.
